# Supplementary material for: Novel Driver Strength Index highlights important cancer genes in TCGA PanCanAtlas patients
Source: PeerJ. 2022 Aug 11;10:e13860. doi: 10.7717/peerj.13860 (PMC9375969; doi:10.7717/peerj.13860)
Supplement: Supplemental Information 2 [file peerj-10-13860-s002.zip › COHORTS/genes plots/detailed/2021_8_16_14_9_distribution_events_detailed_24.pdf]

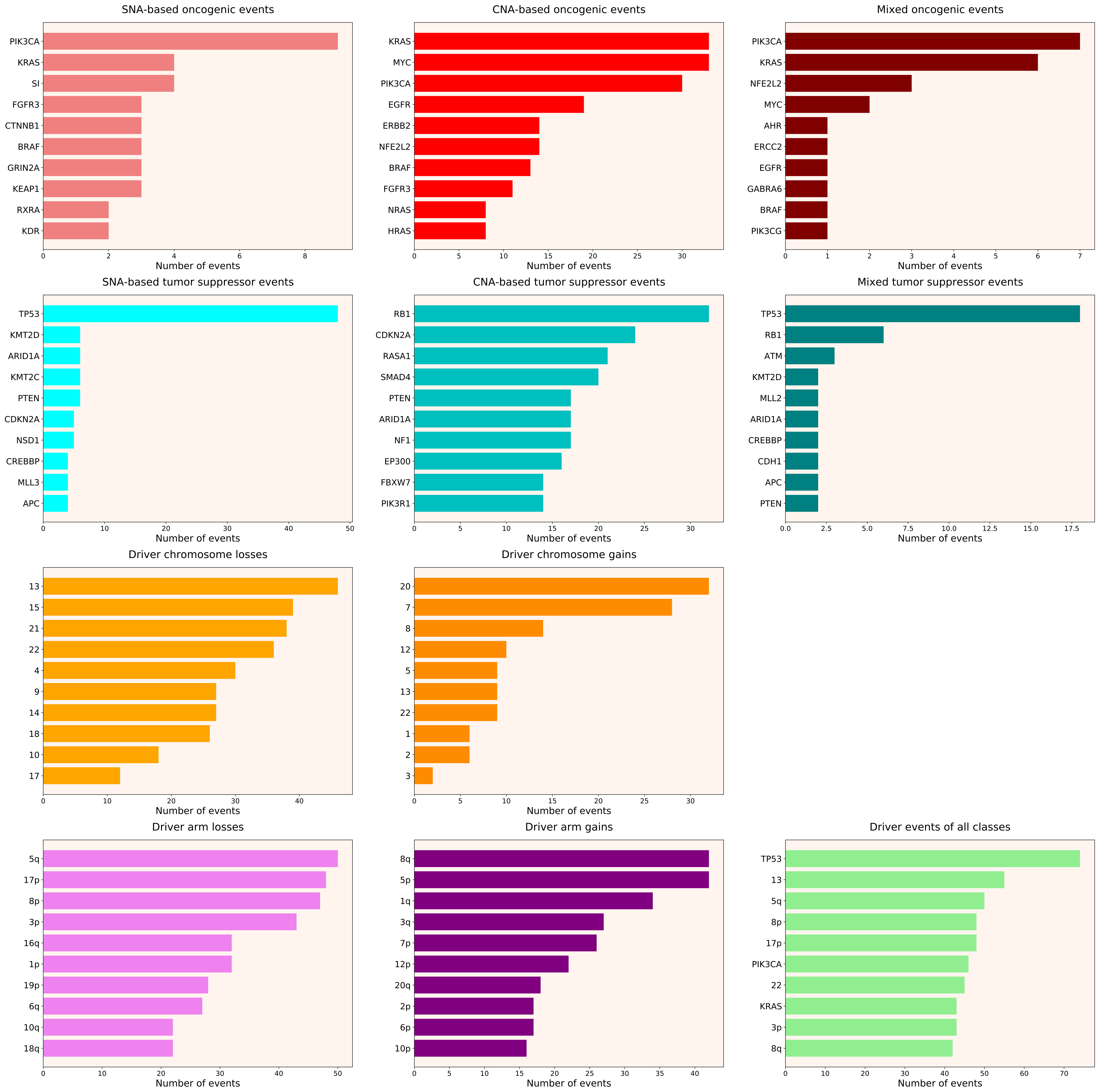
SNA-based oncogenic events
PIK3CA
KRAS
SI
FGFR3
CTNNB1
BRAF
GRIN2A
KEAP1
RXRA
KDR
Number of events
CNA-based oncogenic events
KRAS
MYC
PIK3CA
EGFR
ERBB2
NFE2L2
BRAF
FGFR3
NRAS
HRAS
Number of events
Mixed oncogenic events
PIK3CA
KRAS
NFE2L2
MYC
AHR
ERCC2
EGFR
GABRA6
BRAF
PIK3CG
Number of events
SNA-based tumor suppressor events
TP53
KMT2D
ARID1A
KMT2C
PTEN
CDKN2A
NSD1
CREBBP
MLL3
APC
Number of events
CNA-based tumor suppressor events
RB1
CDKN2A
RASA1
SMAD4
PTEN
ARID1A
NF1
EP300
FBXW7
PIK3R1
Number of events
Mixed tumor suppressor events
TP53
RB1
ATM
KMT2D
MLL2
ARID1A
CREBBP
CDH1
APC
PTEN
Number of events
Driver chromosome losses
13
15
21
22
4
9
14
18
10
17
Number of events
Driver chromosome gains
20
7
8
12
5
13
22
1
2
3
Number of events
Driver arm losses
5q
17p
8p
3p
16q
1p
19p
6q
10q
18q
Number of events
Driver arm gains
8q
5p
1q
3q
7p
12p
20q
2p
6p
10p
Number of events
Driver events of all classes
TP53
13
5q
8p
17p
PIK3CA
22
KRAS
3p
8q
Number of events
